# Horseback Exercise

**Published:** 1854-02

**Authors:** 


### HORSEBACK EXERCISE.

Riding on horseback is, perhaps, of all others, the most manly, elegant and efficient form of exercise. In the first place, it cannot be taken without being out of doors, then it enables you to breathe a larger amount of fresh air than if walking, because you pass through a greater space in less time, and consequently a greater number of layers, or rather sections of fresh air, come in contact with the nostrils, with less fatigue. Another advantage is, that all the muscles of the body are exercised in moderation, and, to a certain extent, equally so. And then again, while thus exercising, and while every step forward gives you a fresh draught of pure out-door air, the mind is entertained by every variety of objects, new things being constantly presented. The only thing to be guarded against is a feeling of chilliness; this is essential, for every chill is an injury; whether a man be sick or well, a chill must necessarily be succeeded by a fever, and fever is disease.

Horseback exercise, to be highly beneficial, should be active, a "hand gallop" or a trot; and, if practicable, a different road should be traveled every day, so that the mind may be diverted by novelties, and thus compelled away from bodily ailments.

The English, as a nation, are a stout, robust, hearty race. The nobility have a long list of names who have lived to the age of seventy, eighty, and even ninety years, but horseback exercise with them is a national amusement; many of them make a ride on horseback as much a matter of course as a daily dinner. Almost the only gentleman seen on horseback in New Orleans is the English merchant, showing the power of a national habit and its influence abroad as well as at home.

If parents could be made to comprehend the full advantages of a constant breathing of pure air to their children, and would be at pains to impress their young minds with its high importance; were they to pay more attention to their physical training, requiring them to take active exercise, for hours every day, on foot and on horseback, there would be some probability that, notwithstanding the heats and impurities of a city atmosphere, those children would grow up in healthfulness, and live to a good old age, instead of paleing away, as they do, long before their prime, growing prematurely old, from a constitution blasted in the bud.

It is owing, mainly, to their delight in out-door exercise, that the elevated classes in England reach a patriarchal age, notwithstanding their habits of high living, of late hours, of wine-drinking, and many other health-destroying agencies; the deaths of their generals, their lords, their earls and their dukes, are chronicled, almost every week, at seventy, eighty, and ninety years; it is because they will be on horseback, the most elegant, rational and accomplished of all forms of mere exercise, both for sons and daughters. But the whole credit of longevity to these classes must not be given to their love of field-sports: it must be divided with the other not less characteristic traits of an English nobleman—*he will take the world easy;* and could we as a people persuade ourselves to do the same thing habitually, it would add ten years to the average of human life, and save many a broken heart, and broken fortune, and broken constitution.

